# MRI based non-invasive detection of cardiomyocyte hypertrophy and cell-volume changes

**DOI:** 10.1186/1532-429X-14-S1-O10

**Published:** 2012-02-01

**Authors:** Otavio R Coelho-Filho, Richard N Mitchell, Heitor Moreno, Raymond Kwong, Michael Jerosch-Herold

**Affiliations:** 1 Internal Medicine, State University of Campinas, Campinas, Brazil; 2 Medicine, Brigham and Women's Hospital, Boston, MA, USA; 3 Radiology, Brigham and Women's Hospital, Boston, MA, USA

## Summary

A new approach has been developed to detect myocardial cell-hypertrophy, by measuring the intra-cellular lifetime of water in a mouse model of hypertensive heart disease, and validating the MRI marker against measurements of cell dimensions on stained heart slices.

## Background

Cardiomyocyte hypertrophy occurs in cardiomyopathies and in response to pressure overload. However, only endomyocardial biopsies allow detection, with the inherent risks of invasive catheter-based procedures. Non-invasive detection of cardiomyocyte hypertrophy using imaging may detect disease at a subclinical stage and potentially guide therapy. To-date, no imaging-technique has been validated to detect hypertrophic response at the cellular level. We developed a novel measure of cell size based on the MRI determination of the intra-cellular lifetime (τic) of water, using pre/post-contrast T1 measurements and a 2-site H-exchange model (2SX-model). We hypothesized that τic correlates positively with the histological measure of cardiomyocyte volume (Vic) in a rodent model of hypertensive heart disease.

## Methods

L-NAME (3mg/ml) or placebo were administered respectively to 17 (bw=37.2±2.3g) and 13 (bw=37.5±2.5g) male-wild-type mice. Mice were imaged at baseline and 7-weeks after treatment on a 4.7T-small-animal MRI-system. T1 (>5T1 measurement/mouse) was quantified with a modified Look-Locker gradient-echo-cine technique, before and after fractionated Gadolinium-DPTA administration. Minor (Dmin) and major (Dmaj) cell-diameters were measured by FITC-labeled wheat germ-agglutinin staining of cell membranes. Morphometric analysis was performed with a computer-based system. Vic was calculated from Dmin and Dmaj cell-diameters using a cylindrical cell-shape approximation.

## Results

**Figure 1:**
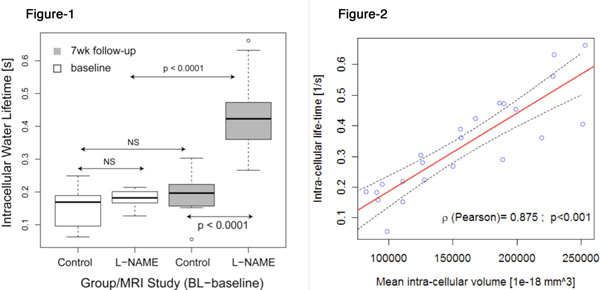
Baseline and 7-week Cell-LifeTIME in control and L-NAME treated-animals. Figure-2: Correlation: intra-cellular volume by histology and Cell-LifeTIME by MRI.

L-NAME-treated-mice developed hypertrophy (weight-indexed LVMass 4.1±0.4 for L-NAME vs. 2.2±0.3μg/g for placebo, p<0.001). Vic (from histology) was substantially higher in L-NAME-treated-animals (19.4*10^3^, IQR 917.1*10^3^μmm^3^ vs. 10.7*10^3^, IQR 9.3*10^3^μmm^3^; p<0.0001), while Dmaj/Dmin was smaller (3.4 vs. 4.2, p<1e-7), compared to controls. τic was significantly higher in L-NAME-treated animals (0.453±0.10 vs. 0.234±0.06, p<0.0001). τic increased significantly from baseline to 7-weeks in animals treated with L-NAME (p<0.0001) (Figure 1). τic strongly correlated with the minor cell diameter (r=0756, P<0.001), Vic (r=0.875, r<0.001) (Figure 1), and more weakly with the major cell-diameter (r=0.478, p=0.02). τic also correlated with weight-indexed LVMass (r=0.71, p<0.001). τic demonstrated an increase from baseline to 7-week (0.177±0.15), which follows the increase of LVmass (39.43±36.6μg/g) in the same interval (r=0.69, p<0.001).

## Conclusions

Quantification of the intra-cellular lifetime of water (τic) by MRI provides a robust non-invasive estimation of cell volume changes, validated here against Vic and direct morphological measurements. τic correlated more strongly with Dmin than Dmaj, reflecting the fact that the dependence τic on Dmax is weak for cylindrical shapes with Dmax/Dmin~4. Dmin was the shape parameter that changes most with hypertension and cell-hypertrophy. This novel MRI-based measure of cell volume may become useful to assess early adverse cellular remodeling in several cardiac conditions.

## Funding

Supported by the American Heart Association (AHA 11POST5550053) and the National Institutes of Health/NHLBI (1R01HL090634-01A1).

